# The impact of pharmaceutical innovation on premature cancer mortality in Canada, 2000–2011

**DOI:** 10.1007/s10754-015-9172-2

**Published:** 2015-06-26

**Authors:** Frank R. Lichtenberg

**Affiliations:** 1 Columbia University, 504 Uris Hall, 3022 Broadway, New York, NY 10027 USA; 2 National Bureau of Economic Research, Cambridge, MA USA

**Keywords:** Cancer, Neoplasm, Mortality, Longevity, Pharmaceutical, Chemotherapy, Innovation, Canada

## Abstract

The premature cancer mortality rate has been declining in Canada, but there has been considerable variation in the rate of decline across cancer sites. I analyze the effect that pharmaceutical innovation had on premature cancer mortality in Canada during the period 2000–2011, by investigating whether the cancer sites that experienced more pharmaceutical innovation had larger declines in the premature mortality rate, controlling for changes in the incidence rate. Premature mortality before age 75 is significantly inversely related to the cumulative number of drugs registered at least 10 years earlier. Since mean utilization of drugs that have been marketed for less than 10 years is only one-sixth as great as mean utilization of drugs that have been marketed for at least a decade, it is not surprising that premature mortality is strongly inversely related only to the cumulative number of drugs that had been registered at least ten years earlier. Premature mortality before age 65 and 55 is also strongly inversely related to the cumulative number of drugs that had been registered at least ten years earlier. None of the estimates of the effect of incidence on mortality are statistically significant. Controlling for the cumulative number of drugs, the cumulative number of chemical subgroups does not have a statistically significant effect on premature mortality. This suggests that drugs (chemical substances) within the same class (chemical subgroup) are not therapeutically equivalent. During the period 2000–2011, the premature (before age 75) cancer mortality rate declined by about 9 %. The estimates imply that, in the absence of pharmaceutical innovation during the period 1985–1996, the premature cancer mortality rate would have increased about 12 % during the period 2000–2011. A substantial decline in the “competing risk” of death from cardiovascular disease could account for this. The estimates imply that pharmaceutical innovation during the period 1985–1996 reduced the number of years of potential life lost to cancer before age 75 in 2011 by 105,366. The cost per life-year before age 75 gained from previous pharmaceutical innovation is estimated to have been 2730 USD. Most of the previously-registered drugs were off-patent by 2011, but evidence suggests that, even if these drugs had been sold at branded rather than generic prices, the cost per life-year gained would have been below 11,000 USD, a figure well below even the lowest estimates of the value of a life-year gained. The largest reductions in premature mortality occur at least a decade after drugs are registered, when their utilization increases significantly. This suggests that, if Canada is to obtain substantial additional reductions in premature cancer mortality in the future (a decade or more from now) at a modest cost, pharmaceutical innovation (registration of new drugs) is needed today.

**Electronic supplementary material:**

The online version of this article (doi:10.1007/s10754-015-9172-2) contains supplementary material, which is available to authorized users.

## Introduction

Previous authors have argued that “reducing premature mortality is a crucial public health objective” (Renard et al. 2014). A widely used measure of premature mortality is years of potential life lost (YPLL) before a given age (e.g. age 75), i.e. the number of years *not *lived by an individual who died before that age (Association of Public Health Epidemiologists in Ontario 2015). Statistics of YPLL are published by the World Health Organization, the OECD, and government agencies of Canada, the U.S., and other countries. Burnet et al. (2005) argue that YPLL “should be considered when allocating research funds.” In the U.S., “cancer [was] responsible for more [YPLL] than all other causes of death combined” in 2008 (National Cancer Institute 2015c). In Canada, premature (before age 75) mortality from cancer is about twice as great as premature mortality from circulatory diseases.

**Fig. 1:**
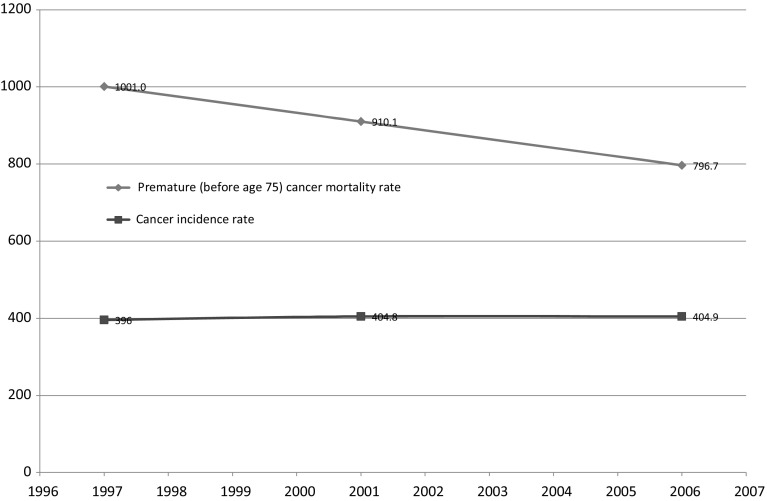
Trends in premature cancer mortality and cancer incidence, Canada, 1997–2006

But as shown in Fig. 1, the premature cancer mortality rate has been declining; it declined about 20 % between 1996 and 2006. The cancer incidence rate remained approximately constant during that period.

**Fig. 2:**
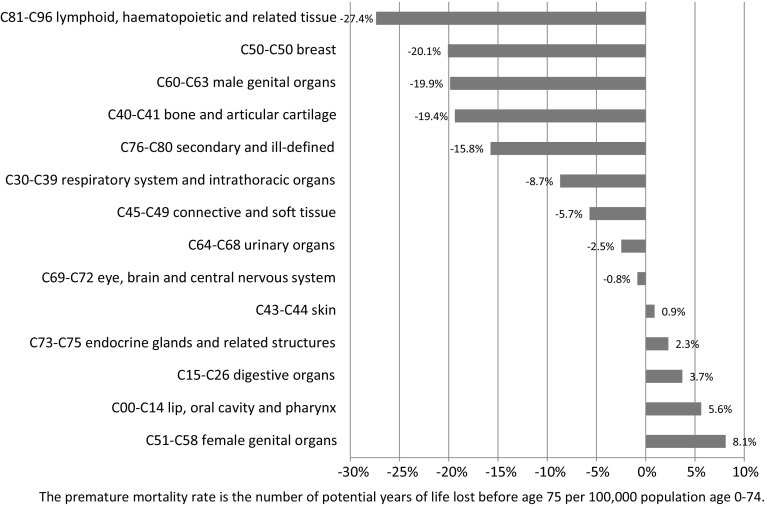
Log change from 2000 to 2011 in the premature mortality rate, by type of cancer, Canada

While the premature mortality rate from all cancers combined has been declining in Canada, Fig. 2 indicates that there has been considerable variation in the rate of decline across cancer sites. During the period 2000–2011, the premature mortality rate from breast cancer declined 20 %, and from cancer of lymphoid, haematopoietic and related tissue declined 27 %, but the premature mortality rate from lip, oral cavity, and pharynx cancer increased 6 %, and from cancer of female genital organs increased 8 %. I will show that this variation in the rate of decline of premature mortality cannot be explained by variation in the rate of decline of incidence.

**Fig. 3:**
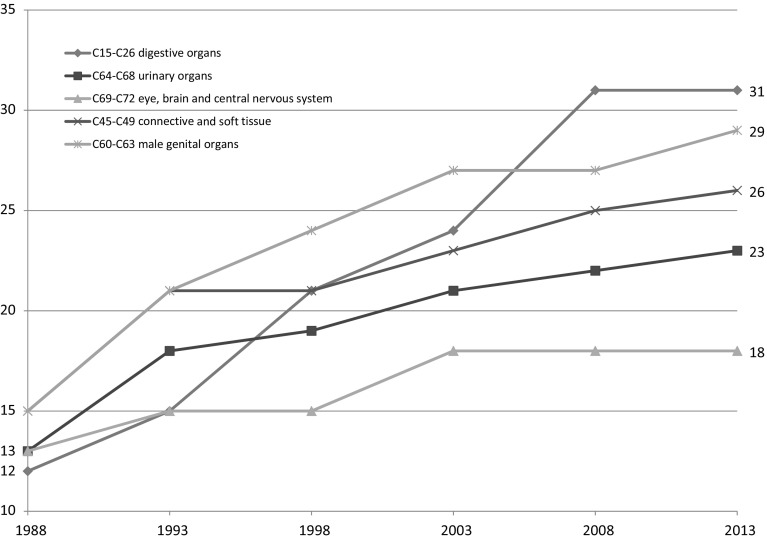
Cumulative number of drugs launched in Canada for 5 types of cancer, 5-year intervals, 1988–2013

In this paper, I will analyze the effect that pharmaceutical innovation has had on premature cancer mortality in Canada during the period 2000–2011.^1^ ${}^{,}$ 2 The analysis will be performed using a difference-in-differences research design based on longitudinal disease-level data. In essence, I will investigate whether the cancer sites that experienced more pharmaceutical innovation had larger declines in the premature mortality rate, controlling for changes in the incidence rate. Figure 3 illustrates that the rate of pharmaceutical innovation, as measured by the number of drugs registered during the period 1988–2013, varied considerably across cancer sites. Only 5 drugs for cancer of the eye, brain and central nervous system were registered, while 19 drugs for cancer of digestive organs were registered.

The analysis will be based on aggregate data—longitudinal data on 15 cancer sites^3^—rather than patient-level data. Stukel et al. (2007) argue that comparisons of outcomes between patients treated and untreated in observational studies may be biased due to differences in patient prognosis between groups, often because of unobserved treatment selection biases. I believe that difference-in-differences estimates based on aggregate panel data are much less likely to be subject to unobserved treatment selection biases than estimates based on cross-sectional patient-level data.^4^ Moreover, the outcome measures that we analyze (premature mortality rates) are not subject to lead-time bias.^5^

In Sect. 2, I describe an econometric model of premature cancer mortality. The data sources used to construct the data to estimate this model are described in Sect. 3. Empirical results are presented in Sect. 4. Key implications of the estimates are discussed in Sect. 5. Section 6 provides a summary and conclusions.

## Premature cancer mortality model

In his model of endogenous technological change, Romer (1990) hypothesized an aggregate production function such that an economy’s output depends on the “stock of ideas” that have previously been developed, as well as on the economy’s endowments of labor and capital. The premature mortality model that I will estimate may be considered a health production function, in which premature mortality is an inverse indicator of health output or outcomes, and the cumulative number of drugs approved is analogous to the stock of ideas. The first model will be of the following form:

1 $$\begin{aligned} \hbox {ln}(\hbox {YPLL75}_{\mathrm{st}})={\upbeta }_{\mathrm{k}} \hbox {CUM}\_\hbox {NCE}_{\mathrm{s,t-k}}+{\upgamma }\, \hbox {ln}(\hbox {INC}\_\hbox {RATE75}_{\mathrm{st}})+{\upalpha }_{\mathrm{s}}+{\updelta }_{\mathrm{t}}+{\upvarepsilon }_{\mathrm{st}} \end{aligned}$$

where, $\hbox {YPLL75}_{\mathrm{st}}$ = years of potential life lost before age 75 from cancer at site s per 100,000 population age 0–74 in year t ($\hbox {t} = 2000, {\ldots }, 2011$); $\hbox {CUM}\_\hbox {NCE}_{\mathrm{i,t-k}}$ $= \sum _{\mathrm{d}}\, \hbox {IND}_{\mathrm{ds}}\, \hbox {REGISTERED}_{\mathrm{d,t-k}}$ = the number of new chemical entities (drugs) to treat cancer at site s that had been registered in Canada by the end of year $\hbox {t}- \hbox {k}$; $\hbox {IND}_{\mathrm{ds}}$ = 1 if drug d is used to treat (indicated for) cancer at site s, 0 if drug d is not used to treat (indicated for) cancer at site s; $\hbox {REGISTERED}_{\mathrm{d,t-k}}$ = 1 if drug d was registered in Canada by the end of year $\hbox {t}-\hbox {k}$, 0 if drug d was not registered in Canada by the end of year $\hbox {t}-\hbox {k}$; INC_RATE75$_{\mathrm{st}}$ = the average annual incidence rate of cancer at site s per 100,000 population age 0–74 in years $\hbox {t}-5, \hbox {t}-4, {\ldots }, \hbox {t}-1$ 6; ${\upalpha }_{\mathrm{i}}$ = a fixed effect for cancer at site s; ${\updelta }_{\mathrm{t}}$ = a fixed effect for year t.

Inclusion of year and cancer-site fixed effects controls for the overall decline in premature cancer mortality and for stable between-disease differences in premature mortality. A negative and significant estimate of ${\upbeta }_{\mathrm{k}}$ in Eq. (1) would signify that diseases for which there was more pharmaceutical innovation had larger declines in premature mortality. The functional form of Eq. (1) has the property of diminishing marginal productivity: the absolute reduction in premature mortality declines with each successive increase in the number of drugs.

**Fig. 4:**
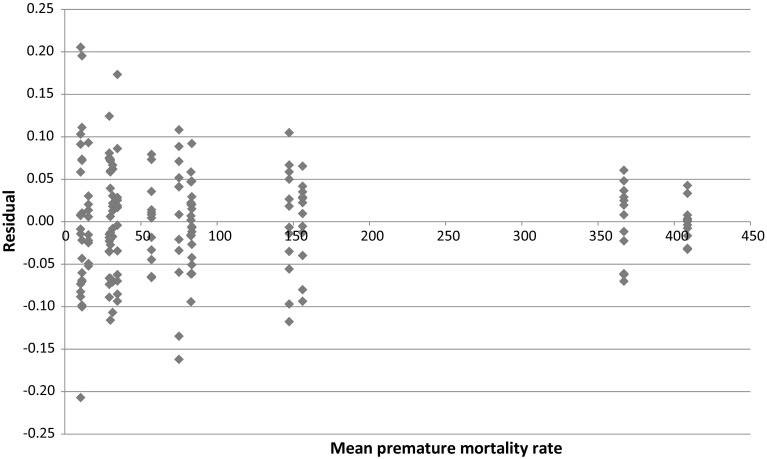
Plot of the residuals from the unweighted regression [ln(YPLL75$_{\mathrm{st}}) = {\upalpha }_{\mathrm{s}}+{\updelta }_{\mathrm{t}}+{\upvarepsilon }_{\mathrm{st}}$] on the mean premature mortality rate $((\sum _{\mathrm{t}} \hbox {YPLL75}_{\mathrm{st}}) / 12)$

As illustrated by Fig. 4, the data exhibit heteroskedasticity—diseases with larger mean premature mortality rates had smaller (positive and negative) annual percentage fluctuations in YPLL75. Equation (1) will therefore be estimated by weighted least-squares, weighting by the mean premature mortality rate during 2000–2011 $((\Sigma _{\mathrm{t}} \hbox {YPLL75}_{\mathrm{it}}) / 12)$. The standard errors of Eq. (1) will be clustered within cancer sites.

Although one would expect an increase in true cancer incidence to increase premature cancer mortality, cancer incidence rates are subject to measurement error, so one should not necessarily expect the coefficient on measured cancer incidence $(\upgamma )$ to be positive. Let I and I* represent measured and true cancer incidence, respectively. Then I = (I / I*) $\times$ I*, and log(I) = log(I / I*) + log(I*). Measured cancer incidence can increase for two reasons: an increase in true cancer incidence, or an increase in the ratio of measured incidence to true incidence. The latter could occur as a result of increasing quantity or quality of cancer screening. More and better cancer screening could lead to earlier diagnosis, which might reduce premature mortality. Therefore the effect on premature mortality of increases in I* and increases in (I / I*) may offset one another: the former is likely to increase premature mortality, but the latter may reduce it. For this reason, although controlling (in an unrestrictive manner) for measured incidence in the premature mortality model seems appropriate, we should not be surprised if we don’t find a significant effect of measured incidence on premature mortality.

Estimation of Eq. (1) enables determination of how much of the decline in Canadian premature mortality during the sample period (2000–2011) can be attributed to the introduction of new drugs. The expression $({\updelta }_{2011}$ – ${\updelta }_{2000})$ indicates the 2000–2011 decline in premature mortality, controlling for (holding constant) the number of drugs and cancer incidence, i.e., in the absence of pharmaceutical innovation. Suppose Eq. (1) is estimated, excluding $\hbox {CUM}\_\hbox {NCE}_{\mathrm{i,t-k}}$, and that the year fixed effects from that equation are denoted by ${\updelta }^{\prime }_{t}$. Then $({\updelta }^{\prime }_{2011}$ – ${\updelta }^{\prime }_{2000})$ indicates the 2000–2011 decline in Canadian premature mortality, not holding constant the number of drugs, i.e., in the presence of pharmaceutical innovation, and $({\updelta }^{\prime }_{2011}$ – ${\updelta }^{\prime }_{2000})$ – $({\updelta }_{2011}$ – ${\updelta }_{2000})$ is an estimate of the 2000–2011 decline in premature mortality attributable to pharmaceutical innovation.

The measure of pharmaceutical innovation in Eq. (1)—the number of chemical substances previously commercialized to treat a disease—is not the theoretically ideal measure. Premature mortality is presumably more strongly related to the drugs *actually* used to treat a disease than it is to the drugs that *could be *used to treat the disease. A preferable measure is the mean *vintage* of drugs used to treat a disease, defined as $\hbox {VINTAGE}_{\mathrm{st}}=\sum _{\mathrm{d}} \hbox {Q}_{\mathrm{dst}} \hbox {LAUNCH}\_\hbox {YEAR}_{\mathrm{d}} / \sum _{\mathrm{d}} \hbox {Q}_{\mathrm{dst}}$, where $\hbox {Q}_{\mathrm{dst}}$ = the quantity of drug d used to treat cancer at site s in year t, and LAUNCH_YEAR$_{\mathrm{d}}$ = the world launch year of drug d.^7^ Unfortunately, measurement of VINTAGE$_{\mathrm{st}}$ is infeasible: even though data on the total quantity of each drug in each year $(\hbox {Q}_{\mathrm{d.t}}=\Sigma _{\mathrm{s}} \hbox {Q}_{\mathrm{dst}})$ are available, many drugs are used to treat multiple diseases,^8^ and there is no way to determine the quantity of drug d *used to treat cancer at site s* in year t.^9^ However, it is shown in Appendix 1 of Lichtenberg (2014a) that there is a highly significant positive correlation across *drug classes* between changes in the (quantity-weighted) vintage of drugs and changes in the number of chemical substances previously commercialized within the drug class.

Pharmaceutical innovation is not the only type of medical innovation that is likely to contribute to premature mortality. Other medical innovation, such as innovation in diagnostic imaging, surgical procedures, and medical devices, is also likely to affect premature mortality. Therefore, measures of these other types of medical innovation should be included in the Eq. (1). Unfortunately, longitudinal disease-level measures of non-pharmaceutical medical innovation are not available for Canada. But failure to control for non-pharmaceutical medical innovation is unlikely to bias estimates of the effect of pharmaceutical innovation on premature mortality, for two reasons. First, pharmaceuticals are more research-intensive than other types of medical care: in 2007, prescription drugs accounted for 10 % of U.S. health expenditure (Center for Medicare and Medicaid Services (2013, Table 2)), but more than half of U.S. funding for biomedical research came from pharmaceutical and biotechnology firms (Dorsey et al. 2010). Much of the rest came from the federal government (i.e. the NIH), and new drugs often build on upstream government research (Sampat and Lichtenberg 2011). The National Cancer Institute (2015b) says that it “has played an active role in the development of drugs for cancer treatment for 50 years... [and] that approximately one half of the chemotherapeutic drugs currently used by oncologists for cancer treatment were discovered and/or developed” at the National Cancer Institute.

Second, previous research based on U.S. data indicates that non-pharmaceutical medical innovation is not positively correlated across diseases with pharmaceutical innovation. In Appendix 2 of Lichtenberg (2014a), it is shown that, in the U.S. during the period 1997–2007, the rate of pharmaceutical innovation was not positively correlated across diseases with the rate of medical procedure innovation and may have been *negatively* correlated with the rate of diagnostic imaging innovation. Also, Lichtenberg (2014b) found that estimates of the effect of pharmaceutical innovation on U.S. cancer mortality rates were insensitive to the inclusion or exclusion of measures of non-pharmaceutical medical innovation. This suggests that failure to control for other medical innovation is unlikely to result in overestimation of the effect of pharmaceutical innovation on longevity growth.

In Eq. (1), premature mortality from cancer at site s in year t depends on the number of new chemical entities (drugs) to treat cancer at site s registered in Canada by the end of year t $-$ k, i.e. there is a lag of k years. Equation (1) will be estimated for different values of k: k = 0, 5, 10, 15, 20, 25.^10^ One would expect there to be a substantial lag because new drugs diffuse gradually—they won’t be used widely until years after registration. Two kinds of evidence—“within molecule” and “between molecule”—support the gradual diffusion hypothesis. The first kind consists of estimates based on the ${\uppi }_{\mathrm{y}}$ parameters from the following equation:

2 $$\begin{aligned} \hbox {ln}(\hbox {SU}_{\mathrm{my}})={\uprho }_{\mathrm{m}}+{\uppi }_{\mathrm{y}}+{\upvarepsilon }_{\mathrm{my}} \end{aligned}$$

where, $\hbox {SU}_{\mathrm{my}}$ = the number of standard units^11^ of molecule m sold in Canada y years after registration (y = 0, 1, ..., 11); ${\uprho }_{\mathrm{m}}$ = a fixed effect for molecule m; ${\uppi }_{\mathrm{y}}$ = a fixed effect for age y

**Fig. 5:**
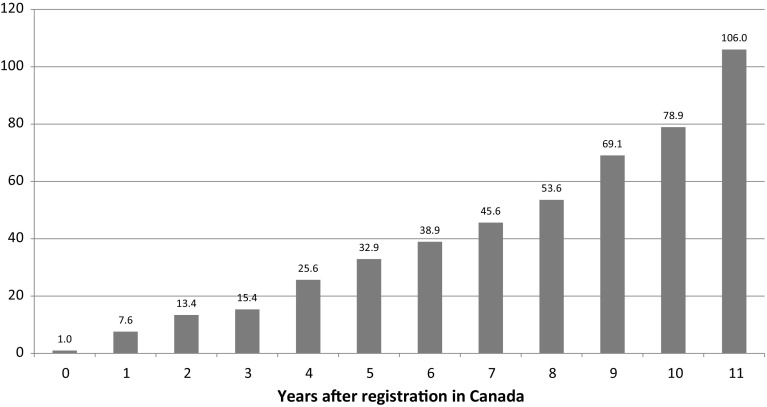
Estimates of the relative utilization index (year 0 = 1.0)

The expression exp$({\uppi }_{\mathrm{y}}-{\uppi }_{0})$ is a “relative utilization index”: it is the mean ratio of the number of units of a molecule sold y years after registration to the number of units of the same molecule sold in the year that it was registered. Using annual data on the number of standard units of molecules sold in Canada during the period 1999–2010, I estimated Eq. (2). Estimates of the “relative utilization index,” based on data on 25 molecules used to treat cancer that were registered after 1998, are shown in Fig. 5. These estimates indicate that the number of units sold 10 years after registration is about ten times as great as the number of units sold one year after registration. Moreover, Fig. 5 provides a conservative estimate of the slope of the age-utilization profile, because there was zero utilization of many of these molecules in the first few years after they were registered.^12^

**Fig. 6:**
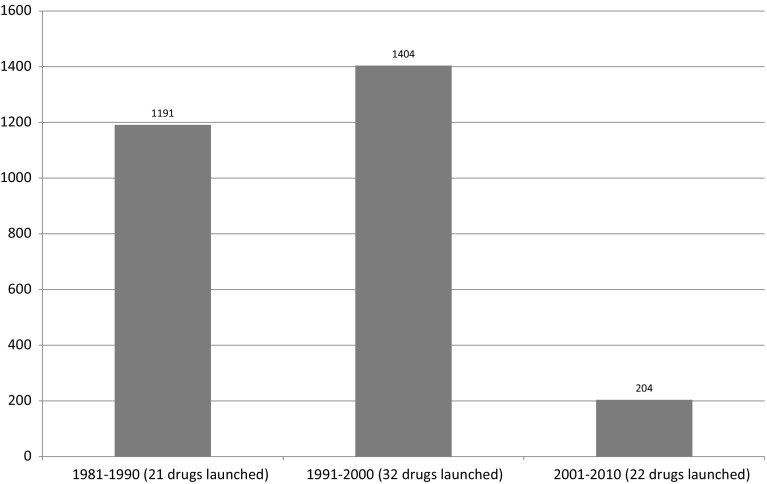
Mean number of standard units of cancer drugs sold (in thousands) in 2010, by period of launch in Canada

Figure 6 provides “between-molecule” evidence of gradual diffusion; it shows data on the mean number of standard units of cancer drugs sold (in thousands) in Canada in 2010, by period of registration in Canada. Mean utilization in 2010 of drugs registered after 2000 is only 15 % as high as mean utilization of drugs registered during 1991–2000, and 17 % as high as mean utilization of drugs registered during 1981–1990.

The relatively low utilization of new drugs may be due to several factors. One is that the prices of old drugs (most of which are no longer patent-protected) are considerably lower than the prices of new, patent-protected drugs. A second factor may be that it takes time for physicians to become knowledgeable about new treatment options. A third potential factor is that new drugs may be targeted at smaller patient populations. Data from the U.S. Food and Drug Administration (2015) indicate that drugs approved by the FDA since 2000 were twice as likely to include pharmacogenomic information in their labeling as drugs approved before 2000. A fourth potential factor is that older drugs are more likely to have supplemental indications, i.e. indications approved after the drug was initially launched, than new drugs.^13^

The measure of pharmaceutical innovation, CUM_NCE$_{\mathrm{s,t-k}}=\sum _{\mathrm{d}}$ IND$_{\mathrm{ds}}$ REGISTERED$_{\mathrm{d,t-k}}$, is based on whether drug d had an indication for cancer at site s *at the end of 2011*. One would prefer to base the measure on whether drug d had an indication for cancer at site s *at the end of year t-k*. FDA data indicate that about one in four new molecular entities has supplemental indications, i.e. indications approved after the drug was initially registered.^14^

In Eq. (1), the measure of premature mortality is the number of years of potential life lost before age 75. This is the age threshold used in Statistics Canada’s key socioeconomic database (CANSIM). Other authorities use different age thresholds; the CDC (2013) provides estimates of YPLL before ages 65, 70, 75, 80, and 85. To assess the robustness of my results, I will estimate models similar to Eq. (1), using age thresholds 65 and 55 as well as 75.

Chemical substances are divided into different groups according to the organ or system on which they act and their therapeutic, pharmacological and chemical properties. In the Anatomical Therapeutic Chemical (ATC) classification system developed by the World Health Organization Collaborating Centre for Drug Statistics Methodology, drugs are classified in groups at five different levels. The highest (1s) level is the “anatomical main group” level; there are 14 anatomical main groups. The 2nd, 3rd, 4th, and 5th levels are “therapeutic subgroup,” “pharmacological subgroup,” “chemical subgroup,” and “chemical substance,” respectively.^15^

Premature mortality from a disease may depend on the number of chemical (or pharmacological) *subgroups* that have previously been developed to treat the disease rather than, or in addition to, the number of chemical *substances* (drugs) that have previously been developed to treat the disease. This will be investigated by estimating versions of Eq. (1) in which CUM_SUBGROUP$_{\mathrm{s,t-k}}$ is included in addition to or instead of CUM_NCE$_{\mathrm{s,t-k}}$, where

$$\begin{aligned} \hbox {CUM}\_\hbox {SUBGROUP}_{\mathrm{s,t-k}}&= \sum _{\mathrm{g}} \hbox {IND}\_\hbox {SUBGROUP}_{\mathrm{gs}}\\&\qquad \times \hbox {REGISTERED}\_\hbox {SUBGROUP}_{\mathrm{g,t-k}}\\ \hbox {IND}\_\hbox {SUBGROUP}_{\mathrm{gs}}&= \hbox {1 if any drugs in chemical subgroup g are}\\&\qquad \hbox {used to treat (indicated for) cancer at site s}\\&= \hbox {0 if no drugs in chemical subgroup g are used to treat}\\&\qquad \hbox {(indicated for) cancer at site s}\\ \hbox {REGISTERED}\_\hbox {SUBGROUP}_{\mathrm{g,t-k}}&= \hbox {1 if any drugs in chemical subgroup g had been}\\&\qquad \hbox {registered in Canada by the end of year t}-\hbox {k}\\&= \hbox {0 if no drugs in chemical subgroup g had been}\\&\qquad \hbox {registered in Canada by the end of year t}-\hbox {k}\\ \end{aligned}$$

## Data

*NCE registrations in Canada (REGISTERED). *Data on new chemical entities registered in Canada were constructed from the Health Canada Drug Product Database, which contains product-specific information on drugs approved for use in Canada. The database is managed by Health Canada and includes human pharmaceutical and biological drugs, veterinary drugs and disinfectant products. It contains approximately 15,000 products which companies have notified Health Canada as being marketed.

*Drug indications (IND).* Data on drug indications were obtained from Thériaque, a database of official, regulatory, and bibliographic information on all drugs available in France, intended for health professionals. This database is produced by the Centre National Hospitalier d’Information sur le Médicament. In this database, drugs are coded according to WHO ATC codes, and diseases are coded according to WHO ICD-10 codes.^16^ The drug indications listed in Thériaque are labeled indications, as defined by the Collège de la Haute autorité de Santé.

Table 1 shows drugs (sorted by registration year) launched after 2006 used to treat various types of cancer in Canada. Appendix Table 1 in supplementary material shows drugs (sorted by registration year) launched since 1951 used to treat various types of cancer in Canada.

**Table 1:** Drugs (sorted by registration year) registered after 2006 used to treat various types of cancer in Canada

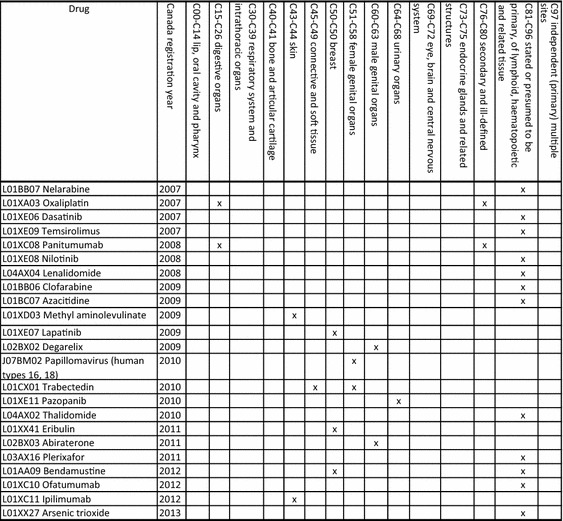	

*Premature mortality data (YPLL75, YPLL65, YPLL55).* Data on years of potential life lost before ages 75, 65, and 55, by cancer site and year (2000–2011), were constructed from the WHO Mortality Database, a compilation of mortality data by age, sex and cause of death, as reported annually by Member States from their civil registration systems.^17^

*Cancer incidence data*. Data on the number of new cancer cases, by cancer site, age, and year, were obtained from CANSIM Table 103-0550.

*Population data*. Data on population, by age and year, were obtained from CANSIM Table 051-0001.

## Empirical results

**Fig. 7:**
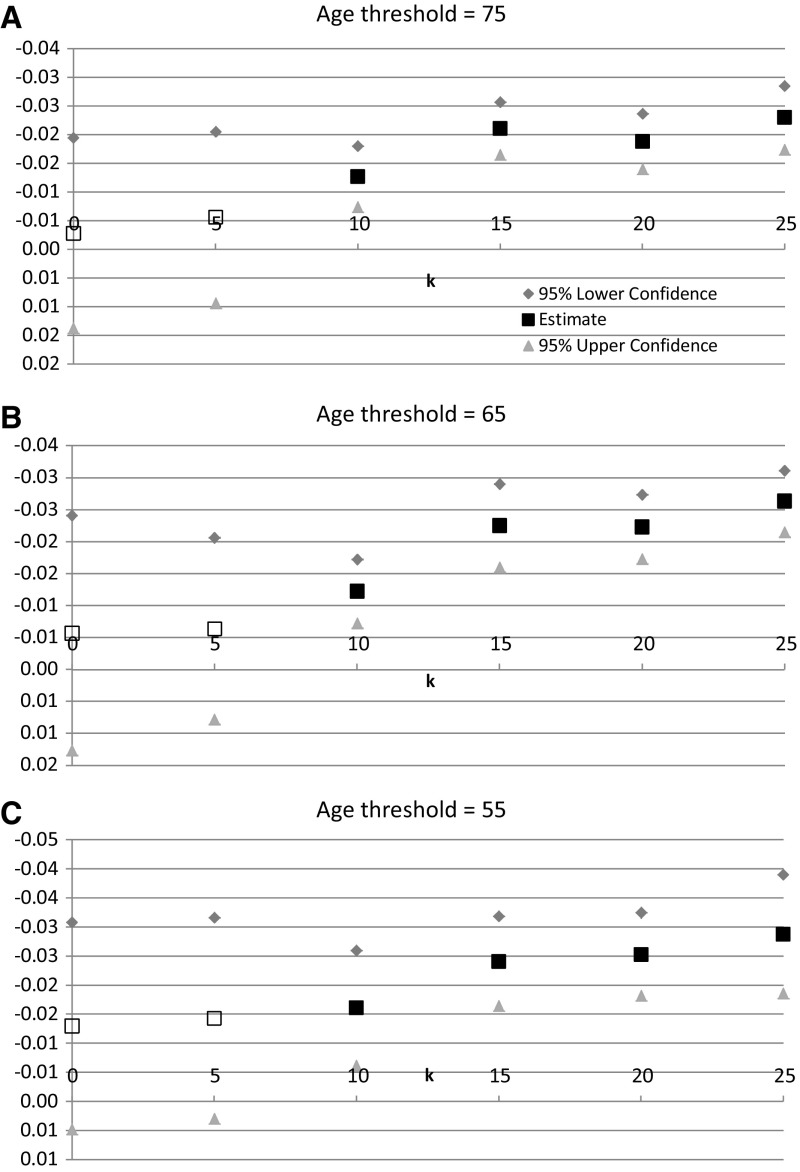
Estimates of the ${\upbeta }_{\mathrm{k}}$ parameters from Eq. (1) and similar equations

Estimates of the ${\upbeta }_{\mathrm{k}}$ parameters from Eq. (1) and similar equations are shown in Table 2 and plotted (on an inverted scale) in Fig. 7. Each estimate was obtained from a separate model. All equations include a cancer incidence measure (e.g. ln(INC_RATE75$_{\mathrm{st}}))$, cancer-site fixed effects and year fixed effects. To conserve space and simplify the presentation, estimates of the cancer incidence coefficient $({\upgamma })$ are not included in Table 2.^18^ None of the estimates of this coefficient were statistically significant, and controlling for cancer incidence had very little effect on the estimates of ${\upbeta }_{\mathrm{k}}$. As discussed earlier, this may be due to offsetting effects of increases in I* and increases in (I / I*) on premature mortality.

**Table 2:** Estimates of the ${\upbeta }_{\mathrm{k}}$ parameters from Eq. (1) and similar equations

Model	Parameter	Lag	Estimate	SE	Z	Pr $>$ ${\vert }\hbox {Z}{\vert }$
A. Dependent variable: ln(YPLL75$_{\mathrm{st}})$ Weight: (($\sum _{\mathrm{t}}$ YPLL75$_{\mathrm{it}})$ / 12)						
1	${\upbeta }_{0}$	0	$-$0.003	0.009	$-$0.33	0.7385
2	${\upbeta }_{5}$	5	$-$0.006	0.008	$-$0.73	0.4659
3	${\upbeta }_{10}$	10	$-$0.013	0.003	$-$4.69	$<$0.0001
4	${\upbeta }_{15}$	15	$-$0.021	0.002	$-$8.96	$<$0.0001
5	${\upbeta }_{20}$	20	$-$0.019	0.003	$-$7.58	$<$0.0001
6	${\upbeta }_{25}$	25	$-$0.023	0.003	$-$8.09	$<$0.0001
B. Dependent variable: ln(YPLL65$_{\mathrm{st}})$ Weight: (($\sum _{\mathrm{t}}$ YPLL65$_{\mathrm{it}})$ / 12)						
7	${\upbeta }_{0}$	0	$-$0.006	0.009	$-$0.61	0.5427
8	${\upbeta }_{5}$	5	$-$0.006	0.007	$-$0.88	0.3784
9	${\upbeta }_{10}$	10	$-$0.012	0.003	$-$4.80	$<$0.0001
10	${\upbeta }_{15}$	15	$-$0.023	0.003	$-$6.78	$<$0.0001
11	${\upbeta }_{20}$	20	$-$0.022	0.003	$-$8.72	$<$0.0001
12	${\upbeta }_{25}$	25	$-$0.026	0.003	$-$10.74	$<$0.0001
C. Dependent variable: ln(YPLL55$_{\mathrm{st}})$ Weight: (($\sum _{\mathrm{t}}$ YPLL55$_{\mathrm{it}})$ / 12)						
13	${\upbeta }_{0}$	0	$-$0.013	0.009	$-$1.43	0.1519
14	${\upbeta }_{5}$	5	$-$0.014	0.009	$-$1.62	0.1045
15	${\upbeta }_{10}$	10	$-$0.016	0.005	$-$3.19	0.0014
16	${\upbeta }_{15}$	15	$-$0.024	0.004	$-$6.10	$<$0.0001
17	${\upbeta }_{20}$	20	$-$0.025	0.004	$-$6.92	$<$0.0001
18	${\upbeta }_{25}$	25	$-$0.029	0.005	$-$5.53	$<$0.0001

Each estimate was obtained from a separate model. All equations include a cancer incidence measure (e.g. ln(INC_RATE75$_{\mathrm{st}}))$, cancer-site fixed effects and year fixed effects. Standard errors are clustered within cancer sites

In part A of Table 2 and Fig. 7, the age threshold for calculating premature mortality is 75 years, i.e. the dependent variable is years of potential life lost before age 75. In model 1, the lag (k) from drug registrations to premature mortality equals zero, i.e. we are examining the effect of the cumulative number of drugs registered by the end of year t on premature mortality in year t. The estimate of ${\upbeta }_{0}$ is not statistically significant. In model 2, the lag is 5 years; the estimate of ${\upbeta }_{5}$ is also statistically insignificant. In models 3–6, the lags are 10, 15, 20, and 25 years, respectively. All of these coefficients are negative and highly statistically significant (p value $<$0.0001), indicating that premature mortality before age 75 is significantly inversely related to the cumulative number of drugs registered at least 10 years earlier. The estimate of ${\upbeta }_{15}$ is the most statistically significant, and the magnitude of the point estimate of ${\upbeta }_{15}$ is 66 % larger than the magnitude of the point estimate of ${\upbeta }_{10}$. Since, as discussed earlier, mean utilization of drugs that have been marketed for less than 10 years is only one-sixth as great as mean utilization of drugs that have been marketed for at least a decade, it is not surprising that premature mortality is strongly inversely related only to the cumulative number of drugs that had been registered at least ten years earlier.

In parts B and C of Table 2 and Fig. 7, the age thresholds for calculating premature mortality are 65 years and 55 years, respectively. The estimates based on these age thresholds are very similar to the estimates based on the age threshold of 75 years: premature mortality before age 65 and 55 is strongly inversely related only to the cumulative number of drugs that had been registered at least ten years earlier.

**Fig. 8:**
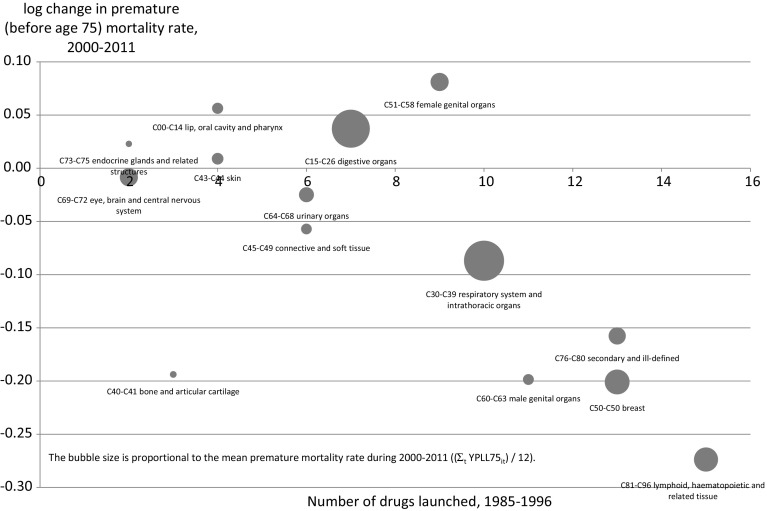
Relationship across cancer sites between the number of drugs launched during 1985–1996 and the 2000–2011 log change in the premature (before age 75 ) mortality rate

Figure 8 shows a bubble plot of the long-run (2000–2011) log change in YPLL before age 75 [ln(YPLL75$_{\mathrm{s,2011}}) -$ ln(YPLL75$_{\mathrm{s,2000}})$] against the long-run change in the cumulative number of drugs registered 15 years earlier [CUM_NCE$_{\mathrm{s,1996}} -$ CUM_NCE$_{\mathrm{s,1985}}$], i.e. the number of drugs registered during the period 1985–1996. The bubble size is proportional to the mean premature mortality rate during 2000–2011 (($\Sigma _{\mathrm{t}}$ YPLL75$_{\mathrm{it}})$ / 12). This figure confirms the finding from model 4 in Table 2 of a highly significant inverse relationship. The point estimate of ${\upbeta }_{15}$ from the long-difference model (${\upbeta }_{15} = -0.0247$, t value = $-$4.42; p value = 0.0010) is similar to the point estimate of ${\upbeta }_{15}$ from model 4 in Table 2.

Figure 8 reveals that the largest number of drugs that were launched during 1985–1996 were for cancers of lymphoid, haematopoietic and related tissue (ICD-10 block C81–C96), which include leukemia. In principle, it is possible that excluding this ICD-10 block could have a substantial effect on the estimates, although it does not appear from Fig. 8 to be an outlier. As shown in Appendix Table 5, when ICD-10 block C81-C96 is excluded from the sample, estimates of Eq. (1) are very similar to the estimates when it is included.

As discussed above, the hypothesis that premature mortality from a disease depends on the number of chemical (or pharmacological) *subgroups* that have previously been developed to treat the disease rather than, or in addition to, the number of chemical *substances* (drugs) that have previously been developed to treat the disease can be tested by estimating versions of Eq. (1) in which CUM_SUBGROUP$_{\mathrm{s,t-k}}$ is included instead of, or in addition to, CUM_NCE$_{\mathrm{s,t-k}}$. Table 3 provides estimates of models suitable for testing this hypothesis. Estimates from three different models are presented there. In all three models, the dependent variable is ln(YPLL75$_{\mathrm{st}})$ and k = 15. The first model shown is the same as model 4 in Table 2, in which the only pharmaceutical variable is CUM_NCE$_{\mathrm{s,t-15}}$. In the second model (model 19), CUM_NCE$_{\mathrm{s,t-15}}$ is replaced by CUM_SUBGROUP$_{\mathrm{s,t-15}}$. The coefficient on this variable is negative and significant, but it is less significant than the coefficient on CUM_NCE$_{\mathrm{s,t-15}}$ in model 4. The third model (model 20) includes both CUM_NCE$_{\mathrm{s,t-15}}$ and CUM_SUBGROUP$_{\mathrm{s,t-15}}$. Controlling for the cumulative number of drugs, the cumulative number of chemical subgroups is not statistically significant. These estimates suggest that drugs (chemical substances) within the same class (chemical subgroup) are not “therapeutically equivalent,”^19^ i.e. they do not have essentially the same effect in the treatment of a disease or condition.

**Table 3:** Estimates of models of years of potential life lost before age 75, including cumulative number of drugs, cumulative number of chemical subgroups, or both Dependent variable: ln(YPLL75$_{\mathrm{st}})$ Weight: (($\sum _{\mathrm{t}}$ YPLL75$_{\mathrm{it}})$ / 12)

Model	Regressor	Estimate	SE	Z	Pr $> {\vert }\hbox {Z}{\vert }$
4	CUM_NCE$_{\mathrm{s,t-15}}$	$-$0.021	0.002	$-$8.96	$<$0.0001
19	CUM_SUBGROUP$_{\mathrm{s,t-15}}$	$-$0.023	0.009	$-$2.55	0.0107
20	CUM_NCE$_{\mathrm{s,t-15}}$	$-$0.024	0.004	$-$6.78	$<$0.0001
20	CUM_SUBGROUP$_{\mathrm{s,t-15}}$	0.010	0.012	0.83	0.4042

All equations include a cancer incidence measure (e.g. ln(INC_RATE75$_{\mathrm{st}}))$, cancer-site fixed effects and year fixed effects. Standard errors are clustered within cancer sites

## Discussion

**Fig. 9:**
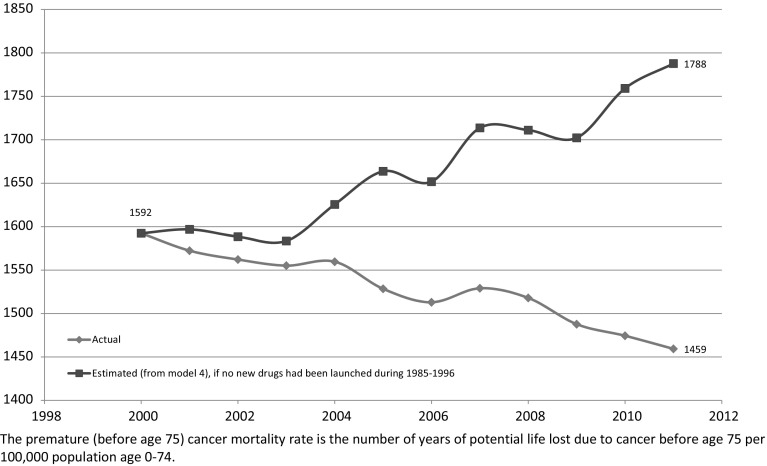
Premature (before age 75) cancer mortality rate: actual versus estimated in the absence of previous pharmaceutical innovation

During the period 2000–2011, the premature (before age 75) cancer mortality rate (the number of years of potential life lost due to cancer before age 75 per 100,000 population age 0–74) declined by about 9 %. The estimates of model 4 imply that, in the absence of pharmaceutical innovation during the period 1985–1996, the premature cancer mortality rate would have *increased *about 12 % during the period 2000–2011.^20^ $$^{,}$$ 21 As shown in Fig. 9, the premature mortality rate would have been 1788, rather than its actual value of 1459. In 2011, the population age 0–74 was about 32.1 million (or 321 hundred thousand), so the estimates of model 4 imply that *pharmaceutical innovation during the period 1985–1996 reduced the number of years of potential life lost to cancer before age 75 in 2011 by 105,366* (= 321 * (1788 – 1459)).

This reduction in premature mortality is an estimate of the *benefit* to Canadians below age 75 in 2011 of pharmaceutical innovation during the period 1985–1996. Now I will calculate an estimate of the (social) *cost* of this innovation. As shown in Appendix Table 1 in supplementary matrial, 40 drugs that are used to treat cancer were registered during the period 1985–1996. Data from IMS Health indicate that in 2010,^22^ expenditure on products containing these molecules was 409 million USD, which is about 1.9 % of total Canadian drug expenditure (21.6 billion USD). About 70 % of cancer patients were diagnosed before the age of 75, so it seems reasonable to assume that 288 million USD (= 70 % * 409 million USD) was spent on these drugs for cancer patients below the age of 75.^23^ This implies that the cost per life-year before age 75 gained from previous pharmaceutical innovation was 2730 USD (= 288 million USD / 105,366 life-years).

Presumably most of the drugs registered during the period 1985–1996 were off-patent by 2010, so these cost estimates reflect prices of generic drugs. Law (2013) argues that Canadian generic drug prices have traditionally been set using a percentage of the equivalent brand-name price as a ceiling, and that typically, these percentages ranged between 60 and 70 % of the brand price.^24^ This suggests that if these drugs had been sold at branded rather than generic prices, the cost per life-year gained would have been between 3900 (= 2730 / 60 %) and 4550 (= 2730 / 70 %) USD. However, the ratio of generic price to branded price may be significantly lower for cancer drugs (which are often infused or injected) than it is for other drugs (which are primarily administered orally). For example, when imatinib, which is used to treat a set of leukemias, went generic in Canada in 2013, the generic drug price was approximately 25 % of the branded price. If the generic/branded price ratio were 25 %, and if these drugs had been sold at branded rather than generic prices, the cost per life-year gained would have been 10,920 (= 2730 / 25 %) USD.

Hirth et al. (2000) performed a search of the value-of-life literature and identified 41 estimates of the value of life from 37 articles.^25^ From estimates of the value of life, they calculated estimates of the value (in 1997 dollars) of a quality-adjusted life-year (QALY).^26^ Four types of methods were used to produce those estimates: revealed preference/job risk, contingent valuation, revealed preference/non-occupational safety, and human capital. Median implied values (in 1997 and 2011 dollars^27^) of a QALY estimated in those studies are shown in the following table.

**Table:** 

Study method	Number of studies	Median value of a QALY	
		1997 dollars	2011 dollars
Revealed preference/job risk	19	$428,286	$600,243
Contingent valuation	8	$161,305	$226,069
Revealed preference/non-occupational safety	8	$93,402	$130,903
Human capital	6	$24,777	$34,725

My estimate of the cost per life-year before age 75 gained from previous pharmaceutical innovation is well below even the lowest estimates of the value of a life-year saved.

## Summary and conclusions

The premature cancer mortality rate has been declining in Canada, and there has been considerable variation in the rate of decline across cancer sites. I analyzed the effect that pharmaceutical innovation has had on premature cancer mortality in Canada during the period 2000–2011, by investigating whether the cancer sites that experienced more pharmaceutical innovation had larger declines in the premature mortality rate, controlling for changes in the incidence rate.

The study is subject to several limitations. First, the measures of pharmaceutical innovation that were used were based only on labeled indications, but the National Cancer Institute (2015d) says that “off-label use of drugs is very common in cancer treatment.” Second, it was not possible to measure or control for non-pharmaceutical medical innovation. Third, the outcome measures used were life-years gained, not quality-adjusted life-years gained.

The estimates indicated that premature mortality before age 75 is significantly inversely related to the cumulative number of drugs registered at least 10 years earlier. Since mean utilization of drugs that have been marketed for less than 10 years is only one-sixth as great as mean utilization of drugs that have been marketed for at least a decade, it is not surprising that premature mortality is strongly inversely related only to the cumulative number of drugs that had been registered at least ten years earlier. Premature mortality before age 65 and 55 is also strongly inversely related to the cumulative number of drugs that had been registered at least ten years earlier. None of the estimates of the effect of incidence on mortality were statistically significant.

Controlling for the cumulative number of drugs, the cumulative number of chemical subgroups does not have a statistically significant effect on premature mortality. This suggests that drugs (chemical substances) within the same class (chemical subgroup) are not therapeutically equivalent.

During the period 2000–2011, the premature (before age 75) cancer mortality rate declined by about 9 %. The estimates imply that, in the absence of pharmaceutical innovation during the period 1985–1996, the premature cancer mortality rate would have increased about 12 % during the period 2000–2011. A substantial decline in the “competing risk” of death from cardiovascular disease could account for this. The estimates imply that pharmaceutical innovation during the period 1985–1996 reduced the number of years of potential life lost to cancer before age 75 in 2011 by 105,366.

The cost per life-year before age 75 gained from previous pharmaceutical innovation is estimated to have been 2730 USD. Most of the previously-registered drugs were off-patent by 2011, but evidence suggests that, even if these drugs had been sold at branded rather than generic prices, the cost per life-year gained would have been below 11,000 USD, a figure well below even the lowest estimates of the value of a life-year gained.

The largest reductions in premature mortality occur at least a decade after drugs are registered, when their utilization increases significantly. This suggests that, if Canada is to obtain substantial additional reductions in premature cancer mortality in the future (a decade or more from now) at a modest cost, pharmaceutical innovation (registration of new drugs) is needed today.

### Electronic supplementary material

Below is the link to the electronic supplementary material.

Supplementary material 1 (xlsx 178 KB)

## Appendix

See Tables 4 and 5.

**Table 4:** Complete estimates of model 4 Dependent variable: ln(YPLL75$_{\mathrm{st}})$ Weight: (($\sum _{\mathrm{t}}$ YPLL75$_{\mathrm{it}})$ / 12)

Parameter	Estimate	SE	Z	Pr $> {\vert }\hbox {Z}{\vert }$
CUM_NCE$_{\mathrm{s,t-15}}$	$-$0.021	0.002	$-$8.96	$<$0.0001
ln(INC_RATE75$_{\mathrm{st}})$	0.064	0.116	0.55	0.5831
Year 2000	$-$0.116	0.032	$-$3.61	0.0003
Year 2001	$-$0.113	0.036	$-$3.16	0.0016
Year 2002	$-$0.118	0.035	$-$3.38	0.0007
Year 2003	$-$0.121	0.029	$-$4.25	$<$0.0001
Year 2004	$-$0.095	0.026	$-$3.68	0.0002
Year 2005	$-$0.072	0.022	$-$3.35	0.0008
Year 2006	$-$0.079	0.019	$-$4.12	$<$0.0001
Year 2007	$-$0.042	0.016	$-$2.60	0.0092
Year 2008	$-$0.044	0.021	$-$2.10	0.0359
Year 2009	$-$0.049	0.022	$-$2.18	0.0290
Year 2010	$-$0.016	0.015	$-$1.08	0.2821
Year 2011	0.000	0.000	–	–
group C00–C14 lip, oral cavity and pharynx	$-$2.154	0.156	$-$13.81	$<$0.0001
group C15–C26 digestive organs	0.285	0.116	2.45	0.0141
group C30–C39 respiratory system and intrathoracic organs	0.567	0.085	6.70	$<$0.0001
group C40–C41 bone and articular cartilage	$-$3.028	0.403	$-$7.52	$<$0.0001
group C43–C44 skin	$-$2.048	0.124	$-$16.47	$<$0.0001
group C45–C49 connective and soft tissue	$-$1.979	0.248	$-$7.98	$<$0.0001
group C50–C50 breast	$-$0.434	0.089	$-$4.90	$<$0.0001
group C51–C58 female genital organs	$-$1.011	0.060	$-$16.87	$<$0.0001
group C60–C63 male genital organs	$-$2.132	0.089	$-$23.94	$<$0.0001
group C64–C68 urinary organs	$-$1.481	0.065	$-$22.75	$<$0.0001
group C69–C72 eye, brain and central nervous system	$-$1.069	0.169	$-$6.31	$<$0.0001
group C73–C75 endocrine glands and related structures	$-$3.277	0.143	$-$22.93	$<$0.0001
group C76–C80 secondary and ill-defined	$-$0.965	0.123	$-$7.82	$<$0.0001
group C81–C96 stated or presumed to be primary, of lymphoid, haematopoietic and related tissue	0.000	0.000	–	–
Intercept	5.730	0.397	14.44	$<$0.0001

Standard errors are clustered within cancer sites

**Table 5:** Estimates of the ${\upbeta }_{\mathrm{k}}$ parameters from Eq. (1) and similar equations, including and excluding C81–C96 lymphoid, haematopoietic and related tissue

Model	Parameter	Lag	Include C81–C96 lymphoid, haematopoietic and related tissue				Exclude C81–C96 lymphoid, haematopoietic and related tissue			
			Estimate	SE	Z	Pr $> {\vert }\hbox {Z}{\vert }$	Estimate	SE	Z	Pr $> {\vert }\hbox {Z}{\vert }$
A. Dependent variable: ln(YPLL75$_{\mathrm{st}})$ Weight: (($\sum _{\mathrm{t}}$ YPLL75$_{\mathrm{it}})$ / 12)										
1	${\upbeta }_{0}$	0	$-$0.003	0.009	$-$0.33	0.7385	0.004	0.008	0.55	0.585
2	${\upbeta }_{5}$	5	$-$0.006	0.008	$-$0.73	0.4659	$-$0.008	0.007	$-$1.06	0.291
3	${\upbeta }_{10}$	10	$-$0.013	0.003	$-$4.69	$<$0.0001	$-$0.012	0.003	$-$3.74	0.000
4	${\upbeta }_{15}$	15	$-$0.021	0.002	$-$8.96	$<$0.0001	$-$0.020	0.003	$-$5.91	$<$0.0001
5	${\upbeta }_{20}$	20	$-$0.019	0.003	$-$7.58	$<$0.0001	$-$0.017	0.006	$-$3.07	0.002
6	${\upbeta }_{25}$	25	$-$0.023	0.003	$-$8.09	$<$0.0001	$-$0.029	0.009	$-$3.18	0.002
B. Dependent variable: ln(YPLL65$_{\mathrm{st}})$ Weight: (($\sum _{\mathrm{t}}$ YPLL65$_{\mathrm{it}})$ / 12)										
7	${\upbeta }_{0}$	0	$-$0.006	0.009	$-$0.61	0.5427	0.006	0.010	0.56	0.579
8	${\upbeta }_{5}$	5	$-$0.006	0.007	$-$0.88	0.3784	$-$0.007	0.008	$-$0.93	0.353
9	${\upbeta }_{10}$	10	$-$0.012	0.003	$-$4.80	$<$0.0001	$-$0.011	0.003	$-$3.43	0.001
10	${\upbeta }_{15}$	15	$-$0.023	0.003	$-$6.78	$<$0.0001	$-$0.019	0.004	$-$5.60	$<$0.0001
11	${\upbeta }_{20}$	20	$-$0.022	0.003	$-$8.72	$<$0.0001	$-$0.017	0.005	$-$3.33	0.001
12	${\upbeta }_{25}$	25	$-$0.026	0.003	$-$10.74	$<$0.0001	$-$0.030	0.009	$-$3.46	0.001
C. Dependent variable: ln(YPLL55$_{\mathrm{st}})$ Weight: (($\sum _{\mathrm{t}}$ YPLL55$_{\mathrm{it}})$ / 12)										
13	${\upbeta }_{0}$	0	$-$0.013	0.009	$-$1.43	0.1519	$-$0.003	0.014	$-$0.18	0.860
14	${\upbeta }_{5}$	5	$-$0.014	0.009	$-$1.62	0.1045	$-$0.013	0.009	$-$1.39	0.166
15	${\upbeta }_{10}$	10	$-$0.016	0.005	$-$3.19	0.0014	$-$0.014	0.004	$-$3.81	0.000
16	${\upbeta }_{15}$	15	$-$0.024	0.004	$-$6.10	$<$0.0001	$-$0.022	0.006	$-$4.05	$<$0.0001
17	${\upbeta }_{20}$	20	$-$0.025	0.004	$-$6.92	$<$0.0001	$-$0.031	0.007	$-$4.49	$<$0.0001
18	${\upbeta }_{25}$	25	$-$0.029	0.005	$-$5.53	$<$0.0001	$-$0.043	0.010	$-$4.15	$<$0.0001

Each estimate was obtained from a separate model. All equations include a cancer incidence measure (e.g. ln(INC_RATE75$_{\mathrm{st}}))$, cancer-site fixed effects and year fixed effects. Standard errors are clustered within cancer sites

## Footnotes

1
Lichtenberg (2014b) analyzed the impact of pharmaceutical innovation and other types of medical innovation on cancer mortality in the U.S. during the period 2000–2009. But as the Squires (2011) demonstrated, the U.S. health care system differs dramatically from the health care systems of other OECD countries, including Canada. For example, in 2008 per capita spending on health was 85 % higher in the U.S. than it was in Canada. Also, the outcome measure and the measure of pharmaceutical innovation used in the present study will differ from those used in Lichtenberg (2014b).

2 I use the sample period 2000–2011 to avoid potential discontinuities in the mortality data, because Canada used the ICD9 cause-of-death classification during 1979–1999, and the ICD10 cause-of-death classification during 2000–2011.

3 The 15 cancer sites are the 15 malignant neoplasm ICD-10 blocks defined by the World Health Organization.

4
Jalan and Ravallion (2001) argued that “aggregation to village level may well reduce measurement error or household-specific selection bias” (p. 10).

5 Survival time for cancer patients is usually measured from the day the cancer is diagnosed until the day they die. Patients are often diagnosed after they have signs and symptoms of cancer. If a screening test leads to a diagnosis before a patient has any symptoms, the patient’s survival time is increased because the date of diagnosis is earlier. This increase in survival time makes it seem as though screened patients are living longer when that may not be happening. This is called lead-time bias. It could be that the only reason the survival time appears to be longer is that the date of diagnosis is earlier for the screened patients. But the screened patients may die at the same time they would have without the screening test. See National Cancer Institute (2015a).

6 The most recent available incidence data are for the year 2010.

7 According to the Merriam Webster dictionary, one definition of vintage is “a period of origin or manufacture (e.g. a piano of 1845 vintage)”. http://www.merriam-webster.com/dictionary/vintage. Robert Solow (1960) introduced the concept of vintage into economic analysis. Solow’s basic idea was that technical progress is “built into” machines and other goods and that this must be taken into account when making empirical measurements of their roles in production. This was one of the contributions to the theory of economic growth that the Royal Swedish Academy of Sciences cited when it awarded Solow the 1987 Alfred Nobel Memorial Prize in Economic Sciences (Nobelprize.org 2015).

8 For example, dactinomycin is used to treat C45–C49 connective and soft tissue neoplasms, C51–C58 female genital organ neoplasms, C60–C63 male genital organ neoplasms, and C64–C68 urinary organ neoplasms.

9 Outpatient prescription drug claims usually don’t show the indication of the drug prescribed. Claims for drugs administered by doctors and nurses (e.g. chemotherapy) often show the indication of the drug. In the US, 70 % of spending on anticancer drugs is for drugs covered under the medical benefit and infused or injected. However, t data on claims for drugs administered by doctors and nurses are not available for Canada.

10 A separate model is estimated for each value of k, rather than including multiple values (CUM_NCE$_{\mathrm{i,t-1}}$, CUM_NCE$_{\mathrm{i,t-2}}$, CUM_NCE$_{\mathrm{i,t-3}}$,...) in a single model because CUM_NCE is highly serially correlated (by construction), which would result in extremely high multicollinearity if multiple values were included.)

11 The number of standard ‘dose’ units sold is determined by taking the number of counting units sold divided by the standard unit factor which is the smallest common dose of a product form as defined by IMS HEALTH. For example, for oral solid forms the standard unit factor is one tablet or capsule whereas for syrup forms the standard unit factor is one teaspoon (5 ml) and injectable forms it is one ampoule or vial. Other measures of quantity, such as the number of patients using the drug, prescriptions for the drug, or defined daily doses of the drug, are not available.

12 Since the dependent variable of Eq. (2) is logarithmic, observations for which SU$_{\mathrm{my}}$ = 0 had to be excluded.

13 The measure of pharmaceutical innovation, CUM_NCE$_{\mathrm{s,t-k}}$ = $\Sigma _{\mathrm{d}}$ IND$_{\mathrm{ds}}$ REGISTERED$_{\mathrm{d,t-k}}$, is based on whether drug d had an indication for cancer at site s *at the end of 2011*. One would prefer to base the measure on whether drug d had an indication for cancer at site s *at the end of year t - k*. Data in the U.S. FDA’s Drugs@FDA data files indicate that about one in four new molecular entities has supplemental indications, i.e. indications approved after the drug was initially launched.

14 Source: Drugs@FDA Data Files.

15 For example, the five levels associated with the chemical subgroup “nitrogen mustard analogues” are:

L: ANTINEOPLASTIC AND IMMUNOMODULATING AGENTS
L01: ANTINEOPLASTIC AGENTS
L01A: ALKYLATING AGENTS
L01AA: Nitrogen mustard analogues
L01AA01: cyclophosphamide
L01AA02: chlorambucil
L01AA03: melphalan
L01AA05: chlormethine
L01AA06: ifosfamide
L01AA07: trofosfamide
L01AA08: prednimustine
L01AA09: bendamustine

16 Many drug databases contain information about drug indications, but this information is usually in text form only.

17 Mortality data are reported in 5-year age groups in the WHO Mortality Database. I assume that deaths in a 5-year age group occur at the midpoint of the age group. For example, I assume that deaths at age 35–39 years occurred at age 37.5. The Association of Public Health Epidemiologists in Ontario (2015) uses this method.

18 Complete estimates of model 4 are presented in Appendix Table 4.

19 According to one medical dictionary, drugs that have “essentially the same effect in the treatment of a disease or condition” are therapeutically equivalent. Drugs that are therapeutically equivalent may or may not be chemically equivalent, bioequivalent, or generically equivalent. http://medical-dictionary.thefreedictionary.com/therapeutic+equivalent.

20 In Appendix Table 4, the point estimate of ${\updelta }_{2000}$ is $-$0.1157 (p value = 0.0003). If CUM_NCE$_{\mathrm{s,t-15}}$ is excluded from the equation, the point estimate of ${\updelta }_{2000}$ is 0.0873 (p value = 0.0008).

21 An important possible reason why the premature cancer mortality rate would have increased in the absence of previous pharmaceutical innovation is a substantial decline in the “competing risk” of death from cardiovascular disease. See Honoré and Lleras-Muney (2006).

22 2010 is the most recent year for which these data are available.

23 Since some of these drugs are used to treat diseases other than cancer, this is probably an overestimate.

24
Law (2013) also argues that recent changes have moved these price ceilings lower in almost every province, to a nationwide low of 25 % in Ontario.

25 Twenty-eight of the reviewed articles used U.S. data; the remaining articles used data from the U.K. (4), Canada (3), France (1), and Denmark (1). National origin did not significantly affect the values.

26 Lichtenberg (2009) demonstrated that, although the health of cancer patients is less than perfect, the number of QALYs gained from pharmaceutical innovation could be either greater than or less than the number of life-years gained.

27 The U.S. Consumer Price Index increased by 40 % between 1997 and 2011.
